# First Nations Peoples’ Eating and Physical Activity Behaviors in Urban Areas: A Mixed-Methods Approach

**DOI:** 10.3390/ijerph191610390

**Published:** 2022-08-20

**Authors:** Anne-Marie Leclerc, Maude Boulanger, Paule Miquelon, Marie-Claude Rivard

**Affiliations:** 1 Department of Nursing, Université du Quebec à Trois-Rivières, Trois-Rivières, QC G9A 5H7, Canada; 2 Department of Human Kinetics, Université du Quebec à Trois-Rivières, Trois-Rivières, QC G9A 5H7, Canada; 3 Department of Psychology, Université du Quebec à Trois-Rivières, Trois-Rivières, QC G9A 5H7, Canada

**Keywords:** First Nations people, perception, lifestyle, health behavior

## Abstract

The dietary transition from traditional to commercial foods and a decrease in physical activity (PA) have impacted the health of the First Nations people of Quebec (Canada), resulting in many suffering from multiple chronic diseases. This study had two objectives: (1) to examine eating and PA behaviors among First Nations peoples in urban areas and (2) to explore the associated health representations. To achieve these objectives, a mixed-methods approach, including a questionnaire (*n* = 32) and a semi-structured interview (*n* = 14), was used to explore the participants’ lifestyle profiles and health experiences. The questionnaire focused on the eating and PA behaviors of First Nations people and their underlying motivations. At the same time, the interviews investigated their health views on diet and PA behaviors based on the conceptual framework of health and its determinants. According to the participants, health is the autonomy to live without pain by maintaining a balance between physical and psychological aspects, eating healthy and exercising. Family and work influence participants’ PA and eating behaviors. Exploring First Nations people’s beliefs and perceptions and the motivations underlying their health behaviors could help encourage the maintenance of a healthy lifestyle despite multiple chronic health conditions.

## 1. Introduction

In Canada, almost 5% of the total population self-identify as Indigenous [1]. The Canadian Constitution recognizes three groups of Indigenous peoples: First Nations (previously called North American Indian), Métis and Inuit [2]. Indigenous people in Canada are diverse, with over 600 communities and more than 70 Indigenous languages [3]. Currently, 56% of First Nations live off-community (reserve) (56%) [3]. A total of 63% of off-community First Nations people report living with one or more chronic conditions (e.g., high blood pressure, arthritis, and asthma), compared to 49% of the total Canadian population [4]. Several health determinants are unfavorable to them, such as smoking, alcohol consumption, obesity, low education, employment, and income [4]. The health issues and social inequities faced by Indigenous communities were exacerbated by the beginning of COVID-19. These issues and inequities include the prevalence of chronic disease (vulnerability to complications of COVID-19), limited access to medical care, inability to respond to sanitation measures (poorly ventilated and overcrowded housing), and economic and psychosocial impacts [5,6].

Specifically in the Province of Quebec, close to 2% of people are Indigenous [1]. Similar to Indigenous communities in other Canadian provinces, they demonstrate significant health differences when compared with the population of Quebec as a whole. A survey on health and well-being among Quebec First Nations people living in a community was published in 2018 [7]. This survey found that most First Nations adults aged 35 and older (59%) suffer from multiple chronic health conditions (e.g., obesity, allergies, and cardiovascular conditions). The prevalence of most of these chronic diseases was stable, except that of obesity, which had increased sharply in adults, from 35% in 2002 to 44% in 2015. In fact, 80% of adults are obese or overweight [8]. Another study about Quebec and Labrador First Nations people living in communities reported that 91% of adults were classified as obese or overweight [9]. While there are many variations (e.g., gender and geography) in the prevalence of obesity among Indigenous communities, statistics for Indigenous people living in urban areas are of concern [10]. In this study, *urban areas* refers to all cities, villages, and other places outside Indigenous communities. Diet and physical activity (PA) play a key role in preventing and managing diseases [11]; hence, these lifestyle factors are examined in this study.

### 1.1. Diet and PA among First Nations People

Among the Indigenous populations of Canada and Quebec, there has been a dramatic increase in chronic diseases due to lifestyle changes [12]. The recommended number of servings in the Canadian Food Guide (First Nations, Inuit, and Métis) for four food groups is as follows: fruits and vegetables (7–8 for women and 7–10 for men), grain products (6–7 for women and 7–8 for men), milk and alternatives (2 for 19–50 years and 3 for over 51 years old), and meat and alternatives (2 for women and 3 for men) [13]. It appears that the current eating behaviors of women First Nations people in Quebec-Labrador fail to meet Canadian recommendations regarding all those food groups [14]. The situation for men is the same, except for grain products [14]. Globally, First Nations people in Quebec-Labrador fail to meet Canadian recommendations regarding the consumption of saturated fat, fiber, sodium, magnesium (men and women aged 31–70), calcium (among women and men aged over 51 years old), and vitamins A, B6 (among women), C (among men and older women and smokers), and D [14]. Over the decades, Quebec Indigenous communities have transitioned from a diet based on hunting, fishing, trapping, and berry picking to a market-oriented diet [12]. Such a transition has led to major health consequences, such as cardiovascular diseases and diabetes [12].

Traditional foods (e.g., moose and beaver) contain protein, vitamins D and B12, niacin, riboflavin, iron, and zinc [12]. In the First Nations Food, Nutrition, and Environment Study conducted in 2016, 95% of adults reported eating traditional food the year preceding the study [14]. However, 84% would like to eat more traditional foods [14]. The main barriers to consuming traditional food are lack of time, availability of hunters in the household, and other resources, such as money, equipment, and transportation [15]. A study conducted on the Cree of northern Quebec associated high consumption of traditional food (three days or more weekly) with participants 40 years and over who were active (who walked 30 min or more each day) and self-identified as hunters [16].

Another reason for the rise in chronic diseases is the increasingly sedentary lifestyle in Indigenous communities, largely due to reduced PA [17,18]. Canadian guidelines recommend that adults engage in at least 150 min of moderate to vigorous aerobic physical activity per week [19]. Researchers estimate that only about one-sixth of First Nations members in Quebec communities are active during leisure [7]. More recent statistics indicate that, among Quebec’s First Nations Peoples living in a community, 71% are sedentary or not very active, while 29% are moderately or highly active [9]. According to a scoping review of the prior 10 years published in 2019, which examined nutrition and PA among Indigenous Canadian peoples, the barriers to PA have been specifically linked to both physical (e.g., climate and lack of infrastructure) and economic (e.g., the relative cost of services) environmental causes [20].

### 1.2. Health Representation

Indigenous health is often described as a holistic concept that includes the whole person [21]. It refers to a balance between the physical (the body, including its physiological homeostasis, healthy eating, and physical activity), mental (intellect), emotional (relational aspects), and spiritual (relationship with the land and with ancestors) dimensions. According to a study conducted in an Atikamekw Nehirowisiw community (Quebec, Canada), language, family relationships, and connections with the territory are the factors that promote wellness [22]. Moreover, according to a Canadian scoping review of diet and PA among Indigenous peoples, there is little theoretical knowledge on motivational aspects [20].

### 1.3. Context and Objective

This study was conducted in the Mauricie and Centre-du-Quebec health regions in the province of Quebec (Canada), especially within two cities concerned by the presence of Indigenous people in urban areas—Trois-Rivières and La Tuque. In Quebec, there is no existing data source that provides an exhaustive portrait of urban First Nations, but nearly 1700 people aged 15 years and over declared First Nations identity in these cities in the 2016 census [23]. Many Indigenous peoples can stay in urban areas for short or long periods. They have several reasons for moving to the city: employment, education, refuge, place of exile, relay between Indigenous communities, or access to health care services [24]. The Atikamekw Nehirowisiwok Nation is the most prevalent. This nation is part of the great Algonquian family [25]. The objectives of this study were (1) to examine eating and PA behaviors among First Nations people in urban areas and (2) to explore the associated health representations.

## 2. Methods

**Figure 1:**
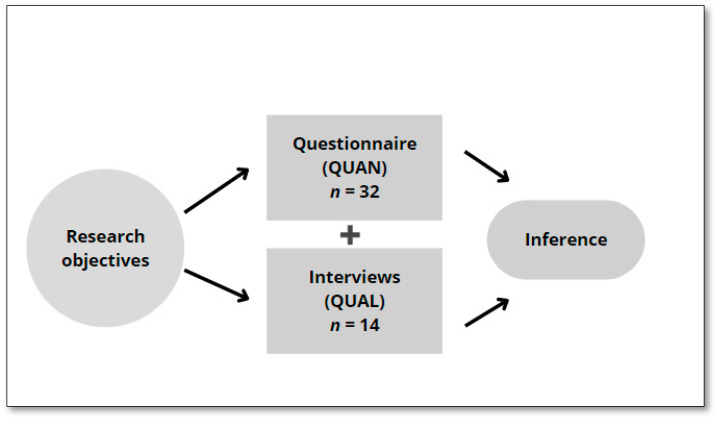
Graphic representation of the mixed design (convergent) used in the study.

A concurrent mixed-methods study was used due to the complementary nature of quantitative (QUAN) and qualitative (QUAL) data [26]. A convergent design was selected (Figure 1); that is, a design that concomitantly combines the results of a qualitative and quantitative analysis during the data collection and analysis stages so that they can be compared or combined [26,27]. This choice is based on the possibility of obtaining a more complete understanding of First Nations peoples’ eating and PA behaviors, in a sense enriching the quantitative results with the qualitative results [28]. Mixed-methods research promotes the use of multiple worldviews or paradigms consistent with First Nations participants [27]. The advantage of using mixed methods is the combination of deductive and inductive approaches [26].

A convenience sampling method was used. The sample consisted of First Nations community members aged 18 years and older. They were recruited between November 2018 and October 2019 via social media (Facebook) and two Native Friendship Centers (Trois-Rivières and La Tuque). The staff of these centers assisted in the recruitment process (e.g., publicity) and facilitated the participation of Elders (knowledge keepers) in the study.

Two data collection methods were used to fulfill the objectives of this study: a questionnaire and individual interviews. Data collection was conducted by the first author. She performed all interviews in a Native Friendship Centres location. Specific recruitment days were scheduled at the Native Friendship Centres and the participants were invited to meet the researcher and complete an online self-administered questionnaire. A card with the researcher’s contact information and web address was given to the participants. At the end of the questionnaire, the participants were asked to leave their contact information if they wanted to participate in the interview. At the same time, an advertisement for the quantitative component of the study was posted on Facebook. Participants were not required to complete either component; some were uncomfortable with the quantitative component. Questions dealing with the participants’ sociodemographic information were compiled for each method. A pilot project was conducted from March 2016 to January 2018 in the Atikamekw Nehirowisiwok and Waban-Aki communities to evaluate the tools used in the study [29]. The pilot project explored a questionnaire on the eating and PA behaviors of Abenaki and Atikamekw Nehirowisiwok Nations members and the meanings associated with these behaviors. A total of 15 participants filled out the questionnaire, and six others participated in the interviews. Based on the results of this pilot project, minor corrections were made to improve the current study (e.g., adding questions on motivation and using different recruitment methods).

### 2.1. Questionnaire

Thirty-two participants completed the questionnaire, which took about 20 min. It included 77 online questions divided into five themes: (1) portrait of nutrition, (2) motivation toward nutrition, (3) portrait of PA habits, (4) motivation toward PA, and (5) sociodemographic information.

(1) To obtain a portrait of nutrition, open-ended questions from the Canadian Food Guide (First Nations, Inuit, and Métis) were used to learn the daily recommendations associated with the four food groups: fruits and vegetables, grain products, milk and alternatives, and meat and alternatives [13]. The questions were inspired by a local questionnaire used in the Waban-Aki community. For example, “How many servings per day, on average, do you get from the following food groups (fruits and vegetables, grain products, dairy products, meat, and alternatives)?”
(2) The questionnaire used to measure participants’ motivation toward nutrition was the Regulation of Eating Behaviors Scale (REBS) [30], which is based on Self-Determination Theory (SDT) [31,32]). The questionnaire contained 24 items (eight items per subscale) divided into five types of motivation: intrinsic (I take pleasure in fixing healthy meals), identified (I believe it will eventually allow me to feel better), introjected (I feel I must absolutely be thin), external (It is expected of me), and amotivation (I don’t know. I can’t see how my efforts to eat healthily are helping my health situation). Participants indicated the extent to which they agreed with each item using a Likert scale ranging from 1 (Does not correspond at all) to 7 (Corresponds exactly).
(3) The questionnaire used to describe participants’ PA practice was the Godin Leisure Time Exercise Questionnaire (GLTEQ), developed by Godin and Shephard (1985). It describes the duration (minutes per week) and frequency of PA practiced at low intensity (e.g., slow walking), moderate intensity (e.g., walking), or high intensity (e.g., running) [33]. The GLTEQ questionnaire is easy to use and reliable [33]. It has been used among Indigenous participants [34,35,36]. Overall, three questions assessed each type of PA intensity on a Likert scale ranging from 1 (Not at all true) to 7 (Completely true) [37].
(4) The questionnaire used to measure motivation toward PA was the Behavioural Regulation in Exercise Questionnaire-2 (BREQ-2) [38]. This questionnaire is also based on SDT [31,32] and includes 19 items divided into five types of motivation: intrinsic (I exercise because it’s fun), identified (I value the benefits of exercise), introjected (I feel guilty when I don’t exercise), external (I exercise because other people say I should), and amotivation (I don’t see why I should have to exercise) (measured on a 5-point Likert scale). Participants indicated the extent to which they agreed with each of the BREQ-2 items using a Likert scale ranging from 0 (Not true for me) to 4 (Very true for me).
(5) Participants’ sociodemographic characteristics, such as gender, age, marital status, education, income, place of residence, and nation of origin, were also measured.

### 2.2. Semi-Directed Interviews

Fourteen participants participated in the interviews, which took about 60 min. The interview guide contained 20 questions grouped according to the four environmental themes presented in the Ministry of Health and Social Services of Quebec’s (MHSSQ) conceptual framework concerning eating habits and PA. Thus, the themes were as follows: (1) the population’s state of health (e.g., definition of health), (2) individual characteristics (e.g., lifestyle habits and behaviors: diet and PA), (3) living environments (e.g., family and community support), and (4) systems (e.g., support from health professionals) [39]. Here is a sample of questions for each theme: (1) What do you think describes a healthy adult? (2) What does PA mean to you? Elaborate on your physical activity habits. (3) How would you rate the support provided by your family for your health in general? About diet and PA? (4) How do you find support from health professionals for healthy eating and PA? Give an example of support that helps and doesn’t help. This framework, described as global and ecosystemic, encourages an in-depth exploration of the meanings associated with eating and PA behaviors. A genogram is a tool that was integrated at the beginning of the interview. It served as an icebreaker for the interview and allowed knowledge of the structure of the family, such as the age and gender of the family members. The health status of all family members was also investigated, allowing for the identification of recurring health problems. The results from the genogram are partially presented in order to preserve the participants’ anonymity. The interview guide was based on the conceptual framework, literature, and previous work on health meanings [40]. Participants’ sociodemographic characteristics, such as gender, age, marital status, education, income, place of residence, and nation of origin, were also measured.

A semi-structured interview was used to gather information to understand the meaning of an event or phenomenon experienced by the participant [41]. Giorgi’s phenomenological research methodology, inspired by Husserl’s philosophical thought, was followed for the collection and analysis of qualitative data [42]. 

The scientific criteria associated with mixed methods refer to the specific criteria for quantitative and qualitative studies. In qualitative research, Lincoln and Guba (1985) suggested five criteria: credibility, dependability, confirmability, transferability, and authenticity [43]. Several strategies have been used to ensure the quality of qualitative data (e.g., triangulation –investigator and method, intercoder checks, obtaining data saturation, and prolonged engagement) [44].

### 2.3. Ethical Considerations

The study respected the OCAP^®^ principles (ownership, control, access, and possession) of the First Nations people [45]. Each participant completed a consent form. The ethics certificate was obtained from the Ethics Review Board of Université du Quebec à Trois-Rivières (CER-18-247-07.04; 27 June 2018).

### 2.4. Analyses

For the quantitative component, descriptive analyses (mean [M] and standard deviations [SD]) of fidelity and validity were performed using SPSS 25.0 software (IBM Corporation, Armonk, NY, USA). For the qualitative component, NVivo 12.0 software (QSR International, Burlington, MA, USA) was used to perform the analyses. Giorgi’s method of phenomenological analysis is descriptive [42]. This method involves five major steps: (1) collecting verbal data, (2) reading (and rereading) the data, (3) dividing the data into meaningful units, (4) organizing and articulating the data from a disciplinary perspective, and (5) synthesizing the data for communication to the scientific community [42]. The codification process was carried out based on the fields in the MHSSQ framework (2012) [39] and subdivided according to the positive or negative influences on the participants’ health. All interviews were analyzed by the first author. Some interviews were selected for codification by the second author to validate the categorization process. The level of agreement between codes was 90% for the entire analysed corpus. Throughout the process, discussions were held with the research team regarding themes emerging from the data coding.

## 3. Results

A complementary inference technique was used to present the results. The qualitative and quantitative results were introduced separately, followed by their respective contributions [46]. The results address both objectives: (1) to examine eating and PA behaviors among First Nations peoples in urban areas and (2) to explore the associated health representations. A total of 32 participants completed the questionnaire (QUAN), and 14 responded to the interview (QUAL).

### 3.1. Results of the Questionnaire (QUAN)

A total of 32 participants (25 women, 7 men) completed the questionnaire (Table 1). The ages of the participants varied widely (M = 40 years old; Min = 20; Max = 70). Most participants were Atikamekw Nehirowisiwok (56.2%) and Waban-Aki (18.8%). Regarding marital status, most were in a relationship or married (56.2%). Regarding employment, most were employed (65.6%). As to the level of education, the percentage of those who had not completed high school (21.9%) or had completed high school (28.1%) was equal to the percentage of those with a university degree (50.0%). The participants’ annual incomes mainly fell into two categories: less than $20,000 (40.6%) and between $20,000 and $39,999 (25.0%).

#### 3.1.1. Portrait of Nutrition

Table 2 presents the number of participants who adhered to the guidelines of the Canadian Food Guide (First Nations, Inuit, and Métis) [8]. Most of the participants consumed the recommended daily portions of meat and its alternatives (90.6%) and milk and its alternatives (81.3%). Compliance with daily recommendations for fruits and vegetables (34.4%) and grain products (12.5%) was less easy.

#### 3.1.2. Motivation toward Eating Behavior

The means shown in Table 3 correspond to the motivations assessed in the REBS questionnaire. The highest mean was found for identified motivation (M = 5.70, SD = 1.20), which was associated with the benefits of healthy eating.

#### 3.1.3. PA Profile

Table 4 presents the average weekly duration of PA in minutes based on intensity level (low, moderate, and high) and accessibility. Low-intensity PA was the most frequent activity among the participants (M = 258, SD = 579). This type of PA was the most accessible (M = 6.6, SD = 0.98). Combining the weekly averages of moderate-and high-intensity PA allowed for evaluating whether participants met the 150-min Canadian recommendation [19]. The results showed that only 37.5% of the participants observed this norm (*n* = 12).

#### 3.1.4. Motivation toward PA

The means shown in Table 5 correspond to each motivation type assessed by the BREQ-2 questionnaire. Intrinsic motivation presented the highest mean (M = 3.16, SD = 0.90) associated with the interest and enjoyment of doing PA.

### 3.2. Results of the Semi-Directed Interviews (QUAL)

A total of 14 participants (Women = 7; Men = 7) participated in the interviews (Table 6). The age of the participants was mainly over 41 years (M = 44.0; Min = 18; Max = 69). The participants were Atikamekw Nehirowisiwok (*n* = 8), Innu (*n* = 4), and Waban-Aki (*n* = 2). Regarding marital status, most participants were not in a relationship (*n* = 9). Regarding employment, most were employed (*n* = 8). Regarding the level of education, the number of participants with a university degree was higher than those who did not have a university degree (*n* = 10). The participants’ annual incomes mainly fell into two categories: less than $20,000 (*n* = 7) and over $20,000 (*n* = 7).

#### 3.2.1. The Population’s State of Health

**Health Perception**. Several physical and psychological pathologies were present in the participants, such as diabetes, anxiety, or chronic pain. However, these pathologies did not necessarily negatively influence participants’ perceptions of their state of health. The following two excerpts demonstrate the divergence in participants’ perceptions when they were asked spontaneously about rating their health on a scale of 0 to 10—a gradation ranging from a perception of poor health to a perception of excellent health:

*At three (on a scale of ten) right now. Yes, it is not great because I work a lot, and I do not do a lot of physical activity. Then, right now, I am not sleeping much because I am working all the time.*

Participant 2 (Atikamekw, Women, 24 years old)

*I would say 9.5 (on a scale of ten). […] I am very disciplined. I get up at 5:00 in the morning to train, and then I work from 8:00 in the morning until 5:00 at night. My meal planning for the week is always done on the weekends. We prepare meals ahead of time to minimize [food] deviations as much as possible.*

Participant 1 (Innu, Women, 47 years old)

**Definition of Health**. Most participants defined health as a balance between the physical (e.g., healthy eating and PA) and psychological (e.g., mental health) aspects of wellness. Some discussed spiritual and emotional aspects (e.g., social networks) as represented by the medicine wheel. Other participants emphasized that it is possible to be healthy despite illnesses or risk factors (e.g., anxiety or being overweight) as long as a person takes care of themselves. In particular, the following participant explained the importance of balance and moderation:

*Someone who is healthy is someone who takes care of himself, psychologically and physically, who pays attention to everything, who never touches drugs, or, maybe, who smokes a cigarette now and then. Who may consume alcohol sometimes, but not too much, not too over the top, in moderation, as they say.*

Participant 7 (Atikamekw, Man, 18 years old)

#### 3.2.2. Individual Characteristics

**Diet.** All participants used strategies to promote healthier eating, such as planning meals, sharing cooking tasks, buying discounted products, varying foods to achieve a balanced diet, drinking water, and taking time to eat with others. The following participant shared his family’s experiences with food:

*Since my wife is sick, I learn things there—not to eat too much poutine [fries, gravy, and cheese]—because she tends to want to order in the restaurants all the time. I tell her, “You can have fries once in a while, but not every day.” I bring either fruit or vegetables. I tell them to eat things with the rest of us—to eat together and then talk about our day.*

Participant 3 (Atikamekw, Man, 68 years old)

However, five participants admitted to not taking time to eat breakfast in the morning. It is worth mentioning that three participants had physical conditions that interfered with appetite (e.g., dental pain or medication with side effects on appetite). The accessibility of food and the high cost of fruits and vegetables were the challenges mentioned. Participants also indicated that, in urban areas, it is difficult to access traditional foods (e.g., moose or beaver). Still, the presence of family members in the communities encourages sharing about once a year. A lack of culinary skills is also a barrier to integrating unfamiliar healthy foods (e.g., eggplants).

**PA.** The most popular PA among the participants was walking. Five participants reported physical (e.g., managing blood sugar in a better way for a person with diabetes), psychological (e.g., providing time for reflection), and spiritual (e.g., helping to get through family difficulties) benefits of PA. However, there are challenges to practicing PA, such as lack of time, motivation, and physical issues. As the following participant pointed out, discrimination against Indigenous people in sports also occurs:

*When I started to work, I neglected this sport, this practice, this way of life, and then slowly, well, the other things took over. The children were born. […] When I was young, I went to the training camp, and then they saw the little “Indian boy” with his bag of equipment arrive, everyone was looking at me.*

Participant 12 (Atikamekw, Man, 49 years old)

#### 3.2.3. Living Environments

**Family Support.** Many participants expressed the supportive role that the family plays in the adoption of healthy lifestyles. In addition to the possibility of sharing tasks within the family, family members influence, motivate, encourage, and model each other. The following excerpt from a participant illustrates this family support as it relates to hockey practice:

*My mom […] is there a lot at my (hockey) games. She tells me things like to shoot when it’s a good time. […] You know, she encourages me a lot. My brother encourages me, too, like he really, really, really encourages me. I love everything he says. He actually inspires me. It’s an inspiration for me not to give up hockey.*

Participant 7 (Atikamekw, Man, 18 years old)

Sometimes, especially when it comes to food, family influence could be negative, as there are some “picky eaters” in the family, and gaps exist in culinary skills:

*My mother always says to us, “I’d like you to cook. But we don’t take the initiative […]. We want to cook, but no, she can’t digest that. For her, it’s too spicy or too fatty, and she can’t digest it. That’s…So we do not take the initiative; it is just too complicated. Makes us lose hope.*

Participant 14 (Waban-Aki, Women, 27 years old)

**Community Support.** As explained by the participants, access to Indigenous cultural knowledge is easier to transmit and receive in communities (e.g., hunting). However, living conditions are difficult over there (e.g., violence and few jobs), as is access to housing. In urban areas, the feeling of belonging to the Indigenous identity is notably conveyed by Native Friendship Centres. They not only organize several cultural activities but also take initiatives related to healthy lifestyle habits (e.g., collective kitchens). However, the participation rate seems to be low at times, as reported by this participant:

*We’re doing more collective kitchens. Then, we really invite people to go there. The world sees this and prefers to stay at home and do nothing. Maybe we should advertise it more. With the Native Friendship Centre, a health walk was organized. There were only two participants.*

Participant 2 (Atikamekw, Women, 24 years old)

**Work Environment.** Some of the workplaces mentioned by the participants had a real influence on adopting healthier eating and increased PA. The following participant said about her culinary discoveries in her workplace:

*Sometimes people here cook. Then it’s really vegetables that I don’t know or haven’t eaten yet. And it’s good when you taste it. It smells good, and that’s it.*

Participant 5 (Atikamekw, Women, 47 years old)

Some employers provide PA opportunities during lunch hours through sports facilities and incentives. Other employers are also sensitive to the well-being of their employees and contribute to their work–life balance. However, employers could do better by providing access to sports facilities or by offering financial incentives (e.g., a discount on gym membership).

#### 3.2.4. Systems

**Health Systems.** Participants who have access to a family doctor or nurse in a clinic have a trusted connection with that healthcare professional. One participant even mentioned being able to reach his doctor directly through a mobile app. Others were not as fortunate to have access to a doctor and spoke of the complexity of the federal and provincial healthcare systems. The following participant compared Indigenous communities to a pen where the management of care differs depending on where one lives:

*If you get sick inside the enclosure (in the community), the [Federal] government will take care of you, treat you, accommodate you, or transport you to a hospital. But if you get sick outside the paddock, you have to manage in another Quebec system […]. So, you become a bit of a hot potato for the government.*

Participant 12 (Atikamekw, Man, 49 years old)

Some participants mentioned their distrust of medication and preferred to use traditional medicine, such as birch, chaga, or the sweat lodge.

**Land Use Planning**. For both food and PA, accessibility remains a major issue in terms of cost (e.g., high fruit and vegetable prices or hockey registration), knowledge of resources (e.g., finding a sports club), and safety (e.g., dangerous roads for pedestrians). The following extract demonstrates this notion of safety in an urban setting:

*Yes, well, there are certainly a lot of areas here that are scary. Like in the area where I live, I don’t go out after eight […]. My area is like a darker part of the city because they often say that there are a lot of drug dealers and not many lights.*

Participant 13 (Innu, Women, 27 years old)

## 4. Discussion

This study’s objectives were (1) to examine eating and PA behaviors among First Nations peoples in urban areas and (2) to explore the associated health representations—were achieved. Although the results were presented according to the measurement instruments used (questionnaire and interviews), the discussion focused on diet and PA behaviors, regardless of these instruments.

### 4.1. Diet

Regarding eating habits, the questionnaire and interview results indicated a discrepancy between the behaviors of First Nations and the recommendations of the Canadian Food Guide (First Nations, Inuit, and Métis). Despite the availability of a food guide to Indigenous people (in French, English, and Indigenous languages), it appeared that the participants had not incorporated all the recommendations into their day-to-day lives. As seen in the community, grain products are not widely consumed [14]. Fruit and vegetable consumption seems low, but the results of the current study reflect an average daily consumption of 6.25 portions (SD = 2.4) per day. In contrast, Canada averages 4.7 portions a day [47]. The Quebec First Nations Regional Health Survey underscores the community’s low fruit and vegetable consumption [7]. It is thus plausible to argue that accessibility to fruits and vegetables is easier in urban areas.

Some participants reported the presence of a “picky eater” in the family as a barrier to healthy eating. Meal preparation becomes a challenge when a second meal must be prepared or a menu change is required to accommodate the “picky eater” [48]. In contrast to the results of Tanguay et al. (2013), who found that young people from an Atikamekw Nehirowisiwok community do not necessarily appreciate traditional food (meat) [49], this reluctance was not directed toward traditional food. Unfortunately, the social and clinical implications of “picky eaters” are still underexplored, especially among adult clients [50], and, to the best of our knowledge, they are not surveyed among Indigenous people.

However, accessibility to traditional foods remains challenging in urban areas, even though they contribute to better nutritional quality [51]. Eating behavior goes well beyond the simple act of eating. For example, being a hunter indicates social status, cultural values, and possession of certain skills and knowledge [16].

All participants in the qualitative component mentioned different strategies to adopt healthier eating behavior (e.g., taking time to eat with others). These results are consistent with the quantitative results, where more prevalent motivation is associated with the benefits of healthy eating (identified motivation). These results suggest that motivation is an important factor to consider in regulating eating behaviors [52]. For example, prior research has shown that intrinsic and identified motivations are positively associated with adopting healthy lifestyle habits and strategies that successfully regulate food intake [29,53,54]. To the best of our knowledge, only two other studies have explored Indigenous motivational factors related to eating behavior [34,55]. However, comparing these results to ours isn’t easy since they are based on the theory of Azjen (1991) and Bandura (1986).

### 4.2. PA

Over half of the questionnaire participants (62.5 %; *n* = 20) failed to meet Canadian recommendations regarding PA, while the interview participants indicated that several obstacles related to finances, accessibility, and human resources contributed to their sedentary behaviors, which was confirmed in the literature [7,56]. The non-Indigenous approach used to quantify PA intensity levels could explain these results. Specifically, some questions of the GLTEQ may have been misinterpreted. A member of the research team was available to complete the questionnaire online with the participants, but some completed it by themselves. For example, the standard deviation for the practice of low-intensity PA was fairly high, as certain participants said they did not engage in any such activity. In contrast, others said they practiced low-intensity PA 120 min daily, seven times a week. According to Paraschak and Thompson (2014) and McHugh (2011), Indigenous peoples do not distinguish between sports, PA, traditional games, and working life [57,58]. However, the GLTEQ was previously used for Indigenous peoples [34,35,36]. Some First Nations people even maintain that the definition of PA includes cultural activity (e.g., drumming) [59]. Moreover, cultural connectedness is a protective factor of PA for First Nations people [35].

As reported by a systematic review of 66 studies conducted among several populations, including populations with chronic illnesses, a very large body of research supports a positive relationship between intrinsic and identified motivation and PA practice [60]. In this study, the most important type of motivation endorsed by the when it comes to PA practice was intrinsic. To the best of our knowledge, no study has yet explored PA motivation among First Nations people.

### 4.3. Diet and PA

Although the level of support from family varied among the participants, support played a significant role in matters of diet and PA practices. According to the Sport for Life Society and Aboriginal Sport Circle (2019), support from family, friends, instructors, volunteers, and coaches is necessary from the playground to the podium [61]. Family support plays a role in physical literacy, which involves motivation, confidence, physical competence, knowledge, and understanding to be active in life [62]. Furthermore, the majority of participants in the qualitative study maintained that the Native Friendship Centers promoted health through curative and preventive treatments. Many activities that encourage healthy eating and PA practice are available in the center and the land. Family and community support are invaluable resources for First Nations people. PA is perceived as collective rather than individual, emphasizing the importance of remaining sensitive to mobilization involving the family and the community.

Health professionals can explore First Nations people’s motivation toward diet and PA practices and promote healthier behaviors. Interventions must consider economic and environmental issues, as well as cultural and historical barriers, such as re-emphasizing the value of traditional foods and encouraging culturally relevant and feasible activities for First Nations peoples [20]. A recent systematic review showed how traditional land-based activities (e.g., subsistence and ceremonial practices) influence Indigenous health and well-being [63]. No comparison was possible between Indigenous communities and urban areas, but holistic wellness was shown in the nine studies. They also reported the importance of Elders, while participants in our study did not discuss this aspect.

### 4.4. Limitations

One limitation of this study concerns the seasonality of diet because data collection took place in the fall—the period when access to fresh produce is difficult. In addition, the quantitative survey did not include any specific questions about traditional foods. It would have been interesting to include a scale measuring the frequency of eating traditional food created by the First Nations Food, Nutrition, and Environment Study [14,15]. Thus, using self-reported measures may have led to an overestimation of PA (and dietary profiles) [64]. This data collection was completed a few months before the start of the COVID-19 pandemic. Therefore, the results presented may be somewhat different from the current context, given the many structural inequalities that have affected and continue to affect the well-being of Indigenous communities (e.g.,food security, funding, and finances) [5,65,66]. Food price inflation is also a concern. For example, Canadians are paying 10% more for food than they were a year ago [67]. Therefore, many families must make choices at grocery stores in terms of food quantity and quality. Finally, one of the strengths of using mixed methods is that it helps answer the research questions effectively, offering the possibility of applying both qualitative and quantitative methodologies. However, despite various recruitment strategies and the collaboration of Indigenous people, the number of participants implicated in the quantitative phase (questionnaire) was small. Some respondents were more interested in the qualitative part (interview) than in the quantitative part (questionnaire). The generalization of the results thus remained limited. Nevertheless, the findings are still relevant because very little is known about urban Indigenous populations.

## 5. Conclusions

In summary, all the results show that the participants were motivated to adopt a healthy diet. In terms of PA, although the benefits were recognized by many participants, motivation to engage in moderate to high intensity physical activity remained relatively low. In accordance with the holistic vision of health of many First Nations and beyond the individual-level factors, all determinants of health have an influence on the adoption of healthy habits, whether at the family, community, health system, or territorial level.

Using a mixed-method inquiry enabled an improved understanding of health from the perspective of Indigenous people. It answered our objectives: (1) to examine eating and PA behaviors among First Nations peoples in urban areas and (2) to explore the associated health representations. At the onset of this project, there was no portrait of modern First Nations diets in Canada [68], and depictions of PA participation were seldom documented [17]. This study thus advanced the exploration of eating behaviors and PA practices among First Nations people. In 2019, the Canadian Food Guide was revised to include Indigenous peoples [69]. The Health Canada Department is currently working with Indigenous partners to support the development of new tools. However, in the meantime, the 2007 version (First Nations, Inuit, and Métis) is still a trustworthy source of information. Eventually, new research tools will be needed to examine eating behaviors in relation to recent Canadian guidelines.

Consistent with the innovative approach proposed in the new version of the Canadian Food Guide [69], work must be done to implement specific new interventions among Indigenous peoples, particularly regarding grain products, fruits, and vegetables. It is important to continue promoting traditional diets within urban areas. First Nations would like a more frequent intake of traditional foods [14]. Indigenous communities have already initiated many projects, such as collective kitchens, recipe books, and community gardens. However, more initiatives (with sustainable funding) are required for urban Indigenous people, such as community refrigerators with traditional food and food baskets. Based on the findings presented, health professionals must consider a holistic perspective when teaching and supporting healthy living. In conclusion, knowledge of the factors that motivate healthy eating and PA practices should be gained to ensure the continuity of healthy initiatives among Indigenous peoples. Further studies are needed to investigate the motivational aspects of healthy lifestyle adoption among Indigenous people.

## Figures and Tables

**Table 1:** Results from the sociodemographic questionnaire—QUAN (*n* = 32).

Variables	*n* (%)
Age (years)	
Younger than 30	11 (34.3)
Between 31 and 40	6 (18.8)
Between 41 and 50	8 (25.0)
Over 50	7 (21.9)
Gender	
Women	25 (78.1)
Men	7 (21.9)
Marital status	
Single (separated, divorced or widowed)	14 (43.8)
In relationship (common law relationship or married)	18 (56.2)
Highest Attained Education	
Elementary school	7 (21.9)
High school diploma	9 (28.1)
College/university degree	16 (50.0)
Income (CAD $)	
<20,000	13 (40.6)
20,000–39,999	8 (25.0)
40,000–59,999	3 (9.4)
60,000–79,999	1 (3.1)
More than 80,000	5 (15.6)
Prefer not to answer	2 (6.3)
Number of children	
None	10 (31.2)
1–2	10 (31.2)
3–4	7 (21.9)
More than 4	5 (15.6)
Job situation	
Unemployed (included sick or pregnancy leave, retired)	7 (21.9)
Student	4 (12.5)
Employed	21 (65.6)
Indigenous origin	
Waban-Aki	6 (18.8)
Algonquian	4 (12.5)
Atikamekw Nehirowisiwok	18 (56.2)
Cree	1 (3.1)
Innu	3 (9.4)

**Table 2:** Compliance with Canadian daily recommendations (*n* = 32).

Compliance with Canadian Daily Recommendations	*n* (%)
Fuits and vegetables	11 (34.4%)
Grain products	4 (12.5%)
Milk and alternatives	26 (81.3%)
Meat and alternatives	29 (90.6%)

**Table 3:** Motivation toward eating behaviors (***n*** = 32).

Type of Motivation	α	M	SD
1. Intrinsic motivation	0.84	4.89	1.56
2. Identified motivation	0.86	5.70	1.20
3. Introjected motivation	0.73	2.94	1.41
4. External motivation	0.79	2.61	1.51
5. Amotivation	0.76	1.91	1.06

Note. α = Cronbach’s alpha; M = mean; SD = standard deviation.

**Table 4:** Average duration (minutes/week) of PA practice and accessibility (*n* = 32).

Weekly PA Practice during Leisure Time According to Intensity	Weekly M in Minutes M (SD)	Average Accessibility M (SD)
Low-intensity PA	258 (579)	6.6 (0.98)
Moderate-intensity PA	104 (154)	5.3 (1.69)
High-intensity PA	46 (79)	4.3 (2.18)

Note. M = mean; SD = standard deviation.

**Table 5:** Portrait of motivation toward PA behavior (*n* = 32).

Type of Motivation	α	M	SD
1. Intrinsic motivation	0.94	3.16	0.90
2. Identified motivation	0.61	2.89	0.67
3. Introjected motivation	0.70	1.69	1.17
4. External motivation	0.86	0.95	0.93
5. Amotivation	0.48	0.48	0.59

Note. α = Cronbach’s alpha; M = mean; SD = standard deviation.

**Table 6:** Results from the sociodemographic questionnaire—QUAL (*n* = 14).

Variables	*n* (%)
Age (years)	
Younger than 30	5 (35.7)
Between 31 and 40	1 (7.1)
Between 41 and 50	4 (28.6)
Over 50	4 (28.6)
Gender	
Women	7 (50.0)
Men	7 (50.0)
Marital status	
Single (separated, divorced or widowed)	9 (64.3)
In relationship (common law relationship or married)	5 (35.7)
Highest Attained Education	
Elementary school	2 (14.3)
High school diploma	2 (14.3)
College/university degree	10 (71.4)
Income (CAD $)	
<20,000	7 (50.0)
20,000–39,999	1 (7.1)
40,000–59,999	4 (28.6)
60,000–79,999	1 (7.1)
More than 80,000	1 (7.1)
Number of children	
None	4 (28.6)
1–2	6 (42.8)
3–4	2 (14.3)
More than 4	2 (14.3)
Job situation	
Unemployed (included sick or pregnancy leave, retired)	5 (35.7)
Student	1 (7.1)
Employed	8 (57.1)
Indigenous origin	
Waban-Aki	2 (14.3)
Atikamekw Nehirowisiwok	8 (57.1)
Innu	4 (28.6)

## Data Availability

The data presented in this study are available on request from the corresponding author. The individual participant data are not publicly available due to ethical requirements.
